# The Effect of Exercise Training on Resting Concentrations of Peripheral Brain-Derived Neurotrophic Factor (BDNF): A Meta-Analysis

**DOI:** 10.1371/journal.pone.0163037

**Published:** 2016-09-22

**Authors:** Adam Dinoff, Nathan Herrmann, Walter Swardfager, Celina S. Liu, Chelsea Sherman, Sarah Chan, Krista L. Lanctôt

**Affiliations:** 1 Neuropsychopharmacology Research Group, Hurvitz Brain Sciences Program, Sunnybrook Research Institute, Toronto, Ontario, Canada; 2 Department of Pharmacology and Toxicology, Faculty of Medicine, University of Toronto, Toronto, Ontario, Canada; 3 Department of Psychiatry, Faculty of Medicine, University of Toronto, Toronto, Ontario, Canada; Cardiff University, UNITED KINGDOM

## Abstract

**Background:**

The mechanisms through which physical activity supports healthy brain function remain to be elucidated. One hypothesis suggests that increased brain-derived neurotrophic factor (BDNF) mediates some cognitive and mood benefits. This meta-analysis sought to determine the effect of exercise training on resting concentrations of BDNF in peripheral blood.

**Methods:**

MEDLINE, Embase, PsycINFO, SPORTDiscus, Rehabilitation & Sports Medicine Source, and CINAHL databases were searched for original, peer-reviewed reports of peripheral blood BDNF concentrations before and after exercise interventions ≥ 2 weeks. Risk of bias was assessed using standardized criteria. Standardized mean differences (SMDs) were generated from random effects models. Risk of publication bias was assessed using funnel plots and Egger’s test. Potential sources of heterogeneity were explored in subgroup analyses.

**Results:**

In 29 studies that met inclusion criteria, resting concentrations of peripheral blood BDNF were higher after intervention (SMD = 0.39, 95% CI: 0.17–0.60, p < 0.001). Subgroup analyses suggested a significant effect in aerobic (SMD = 0.66, 95% CI: 0.33–0.99, p < 0.001) but not resistance training (SMD = 0.07, 95% CI: -0.15–0.30, p = 0.52) interventions. No significant difference in effect was observed between males and females, nor in serum vs plasma.

**Conclusion:**

Aerobic but not resistance training interventions increased resting BDNF concentrations in peripheral blood.

## Introduction

Evidence suggests that physical activity confers cognitive benefits and can alleviate symptoms of psychiatric disorders in many individuals [1–11]. However, the mechanisms through which physical activity confers these benefits have not been fully elucidated. It is likely that a combination of physiological changes induced by physical activity results in these beneficial effects [12–14]. One current hypothesis is that physical activity stimulates the production of brain-derived neurotrophic factor (BDNF), a protein of the neurotrophin family involved in the growth, differentiation, and survival of neurons [12, 15–18]. An increase in BDNF resulting from physical activity is thought to increase adult neurogenesis and synaptogenesis, and prevent neuronal loss, possibly contributing to cognitive benefits and reduced psychiatric symptoms [19–25]. Importantly, studies suggest that peripheral BDNF concentrations may reflect CNS health to some extent, with concentrations typically being lower in patients with psychiatric disorders [26–32] and metabolic disorders [33].

Human and animal studies have examined the impact of physical activity on BDNF concentrations in different body compartments such as blood, muscle, and the brain [34–47]. Animal studies have reported an increase in BDNF after physical activity in various regions of the brain including the hippocampus, prefrontal cortex, motor cortex, lateral septum, cerebellum, striatum, and amygdala [42–56]. Peripheral blood studies in humans have been inconsistent, with some studies reporting increases in BDNF after physical activity and others reporting no significant change or even decreases in BDNF concentrations [20, 40, 57–66]. Possible reasons for this inconsistency include population heterogeneity, differences in type, intensity, and duration of the exercise intervention, and BDNF measurement from different blood components such as serum or plasma [67].

Previous reviews on the effect of exercise on BDNF have highlighted the impact of those study differences [68–71]. A systematic review by Knaepen et al. found a transient increase in peripheral BDNF after acute aerobic exercise but not after resistance training [69]. Furthermore, they concluded that chronic exercise training was unlikely to increase resting concentrations of BDNF but that more research was needed in this area. A review by Zoladz et al. on the same topic found similar results regarding acute exercise but reported mixed results regarding the effect of chronic exercise training on resting peripheral BDNF concentrations [70]. More recently, a systematic review by Huang et al. concluded that both acute and chronic aerobic exercise result in an increase in peripheral BDNF [68]. Consistent with previous reviews, no effect of resistance training on peripheral BDNF concentrations was found. A meta-analysis on the effect of exercise on peripheral BDNF concentrations in humans was published in 2015 by Szuhany et al. [72]. In their analysis, they found a moderate effect size for increases in BDNF following acute exercise and a small but significant increase in resting BDNF concentrations after exercise training. The effect of acute exercise on peripheral blood BDNF concentrations appears to be consistent; however, the effect of chronic exercise training on resting BDNF concentrations is less clear.

Since the last review, more than 20 clinical studies investigating the effect of exercise on peripheral BDNF concentrations have been published, underscoring the importance of this topic. Thus, there is opportunity to re-evaluate the evidence to date and to further investigate moderators of this effect, such as exercise intensity and potential gender differences. This meta-analysis of human studies sought to quantify the magnitude and consistency of the effect of exercise training on resting concentrations of BDNF in peripheral blood. Potential differences in this effect across gender, age, and blood component (i.e. serum vs plasma) were also examined. Furthermore, the impacts of exercise modality, intensity, and duration on this effect were examined as possible sources of heterogeneity.

## Methods

### Data Sources

The Preferred Reporting Items for Systematic Reviews and Meta-Analyses (PRISMA) guidelines [73] were followed for this meta-anlysis (S1 Text). English-language literature was searched using MEDLINE, Embase, PsycINFO, SPORTDiscus, Rehabilitation & Sports Medicine Source, and Cumulated Index to Nursing and Allied Health Literature (CINAHL) databases. One non-English language study was included (Koichiro et al. 2015), as a translator was available to extract information from that study. Databases were searched up to February 2016 for original reports of BDNF changes after exercise. A sample search strategy (MEDLINE) is presented in S2 Text. Reference lists of retrieved studies were searched for additional reports.

### Study Selection

Inclusion criteria were as follows: 1) measured serum, plasma, or whole blood BDNF concentration; 2) BDNF measured before and after an exercise intervention; 3) intervention ≥ 2 weeks; 4) exercise intensity ≥ 50% of peak oxygen uptake (VO_2Peak_), or if exercise intensity was not reported, exercise described as running, cycling, or resistance training. Exclusion criteria were as follows: 1) study included a diseased population (e.g. diabetes, Parkinson’s disease, multiple sclerosis, etc.) or a psychiatric population (e.g. depression, schizophrenia, etc.); 2) study population consisted of children below the age of 18; or 3) study had significant co-interventions likely to impact the effect of exercise on BDNF concentrations (e.g. military training [74], restricted sleep, etc.) as these groups have altered BDNF concentrations which may modify the effect of exercise training on BDNF concentrations [33, 75–79]. Some studies that met these initial eligibility criteria were not included in this meta-analysis as the data were not extractable (e.g. standard deviation not reported) or the exercise intensity description implied that the intervention intensity was < 50% VO_2Peak_ (e.g. yoga or easy walking).

### Data Extraction

Two independent raters examined each article for eligibility. Disagreements regarding inclusion were settled by consensus with a third rater. Data on pre- and post-intervention mean BDNF concentrations and standard deviations [picograms/millilitre], population characteristics, exercise intervention characteristics, risk of bias items, and other study details were extracted into a pre-formatted spreadsheet by two raters. Missing data were requested from the corresponding authors. Exercise intensity prescriptions for percentage of maximum heart rate were converted to percentage of maximum VO_2Peak_ as described by the National Council on Strength & Fitness [80]. In studies with multiple exercise intervention groups, groups were combined for the overall analysis. Studies were additionally categorized by whether the exercise intervention was aerobic or resistance training. In studies with an exercise intervention consisting of both aerobic and resistance training, interventions in which >50% of the time was spent performing aerobic exercise were considered aerobic and vice-versa.

### Statistical Analyses

Standardized mean differences (SMD) and 95% confidence intervals (CI) were calculated using random-effects models [81]. SMDs were chosen because of variability in absolute BDNF concentrations between assays used by different laboratories and between measures of BDNF in different components of blood [82]. Random-effects models are preferred if significant heterogeneity is expected, as they account for variable underlying effects in estimates of uncertainty, including both within- and between- study variance. Heterogeneity across studies was summarized by Q statistics calculated in Chi-square analysis and I^2^ indices were calculated to investigate inconsistencies among results of the included studies [83]. Heterogeneity was further explored via subgroup analysis. In one study that measured BDNF in both serum and plasma [41], serum measurements were used in all analyses except subgroup analysis of serum versus plasma (in which both measurements were used), as serum BDNF measurements were more common across studies than plasma measurements. Inverse variance-weighted meta-regression analyses were used to investigate associations between SMD and population characteristics and intervention characteristics. Risk of publication bias was assessed visually using funnel plots and quantitatively with Egger’s test [84]. Study quality was assessed using criteria adapted from the Cochrane Collaboration’s Risk of Bias tool as done previously [85]. Analyses were conducted using Review Manager Version 5.3 (Cochrane Collaboration, Oxford, UK) and Stata (Release 14.1; StataCorp, College Station, TX).

## Results

### Population Characteristics

**Fig 1:**
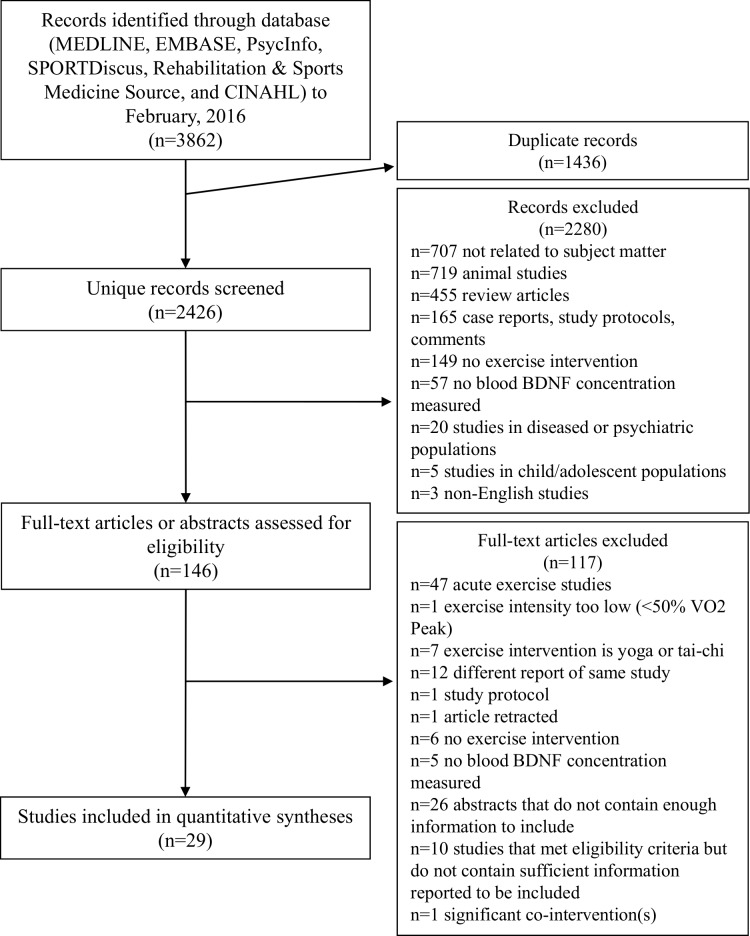
Search and selection of articles.

Twenty-nine studies met inclusion criteria and presented sufficient data to be included in this meta-analysis (Fig 1). Disagreement on whether a study met inclusion criteria arose for one study, and consensus was reached. Reasons for exclusion are shown in Fig 1. 910 participants (61.3% male, mean age 42.2 ± 22.4, mean BMI 25.8 ± 2.3) were included. Included studies ranged in size from 7 to 304 participants (Table 1) completing an exercise intervention. Mean exercise prescriptions were 50.9 ± 16.3 (20–90) minutes for 3.1 ± 1.0 (2–7) sessions per week for 12.4 ± 8.7 (5–52) weeks (Table 2).

**Table 1:** Baseline study population characteristics.

Author, year	n	Gender (% male)	Mean age (years)	Mean BMI (kg/m^2^)	Mean VO2 Peak (mL∙kg^-1^∙min^-1^)
Araya et al [41], 2013	15	40	38.3	30.6	?
Babaei et al [86], 2013	22	100	55.7	27.6	?
Bos et al [87], 2013	24	37.5	32.1	24	38.3
Cho et al [88], 2014	23	0	42.7	23.4	29.4
Coelho et al [89], 2012	20	0	71.5	29.7	?
Damirchi et al [58], 2014	11	100	54.1	29.8	28.4
Erickson et al [20], 2011	60	27	67.6	?	21.4
Ferris et al [90], 2006	18	16.7	20	?	?
Forti et al [60], 2014	19	55	65.7	24.7	?
Forti et al [91], 2015	49	49	68	27.1	?
Fragala et al [92], 2014	13	?	?	?	?
Gapin et al [93], 2013	20	40	51.4	30.2	?
Goekint et al [61], 2010a	15	80	20.1	23.9	?
Goekint et al [94], 2010b	9	?	21.2	23.2	?
Kim H et al [95], 2015	66	0	81.1	?	?
Kim Y [96], 2015	7	100	20.6	21.8	?
Koichiro et al [97], 2015	12	100	35	24.6	24.5
Lemos et al [98], 2016	304	100	24.3	24.8	?
Levinger et al [99], 2008	23	48	50.7	28.5	?
Mueller et al [100], 2015	16	43.8	27.2	33.6	?
Murawska-Cialowicz et al [101], 2015	12	58.3	25.6	24.2	38.3
Prestes et al [102], 2015	39	0	67.4	27.9	?
Ruiz et al [103], 2015	20	20	92.3	25.5	?
Schiffer et al [104], 2009	18	?	22.4	?	?
Seifert et al [105], 2010	7	100	29	27.3	?
Wagner et al [106], 2015	17	100	25	23.8	45.9
Williams & Ferris [107], 2012	18	16.7	20	21.9	33.8
Yarrow et al [108], 2010	20	100	21.9	25.9	?
Zoladz et al [109], 2008	13	100	22.7	23.7	45.3

? indicates uncertain

**Table 2:** Exercise intervention characteristics of included studies.

Author, year	Duration (weeks)	Frequency (sessions per week)	Intervention	Intensity	Session time (mins)	Modality
Araya et al [41], 2013	10	3	Exercise	≥ 65% VO2 Peak	60	running or cycling
Babaei et al [86], 2013	6	3	Exercise	50–60% VO2 Peak	60	walking and running
Bos et al [87], 2013	12	3	Exercise	?	29.5	walking and running
Cho et al [88], 2014	24	4	Exercise	Aerobic training group: 50–80% VO2 Peak; Combined exercise group:?	60	Resistance training, ab exercises, running, jogging
Coelho et al [89], 2012	10	3	Exercise	50–75% of 1RM, 8 reps for each exercise	60	resistance training
Damirchi et al [58], 2014	6	3	Exercise	50–60% VO2 Peak	62.5	walking and running
Erickson et al [20], 2011	52	3	Exercise	Weeks 1–7: 50–60% HR Max Weeks 8–52: 60–75% HR Max	40	walking
Ferris et al [90], 2006	12	3	Exercise	65–75% VO2 Peak	30	running
Forti et al [60], 2014	12	3	Exercise	50–80% of 1RM, 10 reps x 3 sets for each exercise	60	resistance training
Forti et al [91], 2015	12	3	Exercise	6 on the OMNI scale of perceived exertion	37.5	resistance training
Fragala et al [92], 2014	6	2	Exercise	5 or 6 on the OMNI scale of perceived exertion	?	resistance training
Gapin et al [93], 2013	13	?	Exercise, Dietary Restrictions	?	?	?
Goekint et al [61], 2010a	10	3	Exercise	50–80% of 1RM, 10 reps x 3 sets for each exercise	45	resistance training
Goekint et al [94], 2010b	8	3	Exercise	?	30	walking, running, or cycling
Kim H et al [95], 2015	13	2	Exercise, One group given milk fat globule membrane supplements	12–14 RPE on the Borg Scale	60	resistance training
Kim Y [96], 2015	8	5	Exercise	11–15 RPE on the Borg Scale	80	running and taekwondo
Koichiro et al [97], 2015	16	2	Exercise	High intensity interval training; >90% VO2 peak separated by periods of 60 sec active rest	20	cycling or arm and leg ergometer
Lemos et al [98], 2016	17	3	Exercise	80% VO2 Peak	90	jogging and running, body weight exercises
Levinger et al [99], 2008	10	3	Exercise	Week 1: 40–50% of 1RM, 15–20 reps x 2 sets for each exercise; Week 2–10: 50–85% of 1RM, 8–20 reps x 3 sets for each exercise	55	resistance training
Mueller et al [100], 2015	13	2	Exercise	70–80% HR Max	60	resistance training
Murawska-Cialowicz et al [101], 2015	13	2	Exercise	85–95% HR Max	60	crossfit
Prestes et al [102], 2015	16	2	Exercise	?	45	resistance training
Ruiz et al [103], 2015	8	3	Exercise	Aerobic training group: 10–12 RPE on Borg Scale; Resistance training group: 30–70% of 1RM, 8–10 reps x 2–3 sets for each exercise	42.5	cycling and resistance training
Schiffer et al [104], 2009	12	3	Exercise	Aerobic training group: 50–60% HR Max; Resistance training group: 70–80% of 1RM, 8–10 reps x 3 sets for each exercise	45	resistance training and running
Seifert et al [105], 2010	12	7	Exercise, Dietary Restrictions	70% HR Max or 65% VO2 Peak	60	cycling, running, swimming, or rowing
Wagner et al [106], 2015	6	3	Exercise	77% VO2 Peak	60	cycling
Williams & Ferris [107], 2012	12	3	Exercise	65–70% HR Max	30	jogging
Yarrow et al [108], 2010	5	3	Exercise	70% of 1RM, 6 reps for each exercise	?	resistance training
Zoladz et al [109], 2008	5	4	Exercise	90% of VO2 at Lactate Threshold	42.5	cycling

? indicates uncertain

### Comparison of Pre- and Post-Intervention BDNF Concentration

**Fig 2:**
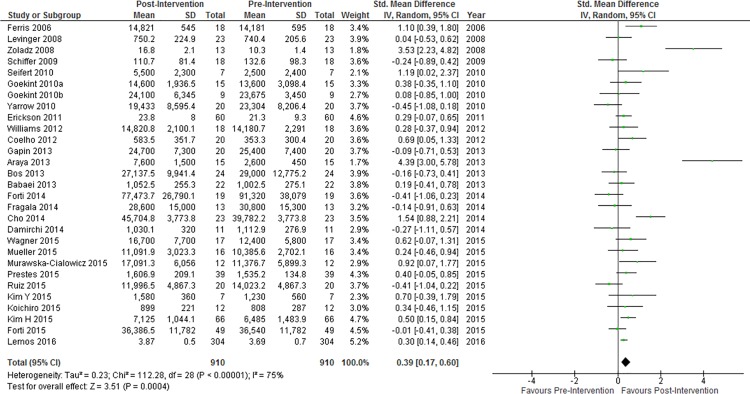
Resting peripheral BDNF concentration change pre- to post-intervention. Diamond indicates SMD and 95% CI. Significance of overall effect: Z = 5.00, P < 0.00

Resting concentrations of peripheral blood BDNF were significantly higher after an exercise training intervention (Fig 2). Neither a Funnel plot (S1 Fig) nor Egger’s test (p = 0.18) revealed significant risk of small-study effects. A Q^2^ value of 112.28 and I^2^ index of 75% signify considerable heterogeneity and inconsistency, respectively, among included studies. Qualitatively, nine of twenty-nine studies (31.0%) reported a significant increase in resting peripheral BDNF concentrations, no studies reported a significant decrease in resting peripheral BDNF concentrations, and twenty studies (69.0%) reported no significant change. Twenty-eight of the twenty-nine included studies were deemed to have high methodological quality (S1 Table).

### Investigations of Heterogeneity

#### Aerobic Exercise vs Resistance Training

**Fig 3:**
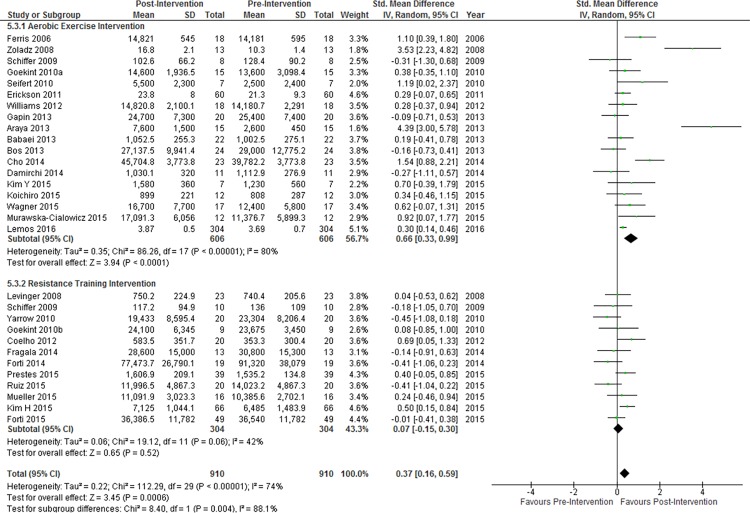
Subgroup analysis of aerobic exercise interventions vs resistance training interventions. Diamonds indicate SMD and 95% CI. Test for subgroup differences: P = 0.004.

In eighteen studies in which the exercise intervention consisted of entirely or mostly (>50% of the time) of aerobic exercise, a greater increase in resting peripheral BDNF concentration was found (SMD = 0.66, 95% CI: 0.33–0.99, p < 0.001) than that found when all studies were combined. Substantial heterogeneity and inconsistency were present in this subgroup (Q^2^ = 86.26, I^2^ = 80%). In contrast, in twelve studies in which the exercise intervention consisted entirely or mostly of resistance training, there was no change in resting peripheral BDNF concentration after exercise (SMD = 0.07, 95% CI: -0.15–0.30, p = 0.52, Q^2^ = 19.12, I^2^ = 42%). The difference in effect size between these two subgroups was significant (Fig 3). Of note, one study [104] included both an aerobic exercise group and a resistance training group and these separate groups were included in their respective subgroups.

#### Other Exercise Intervention Characteristics

**Fig 4:**
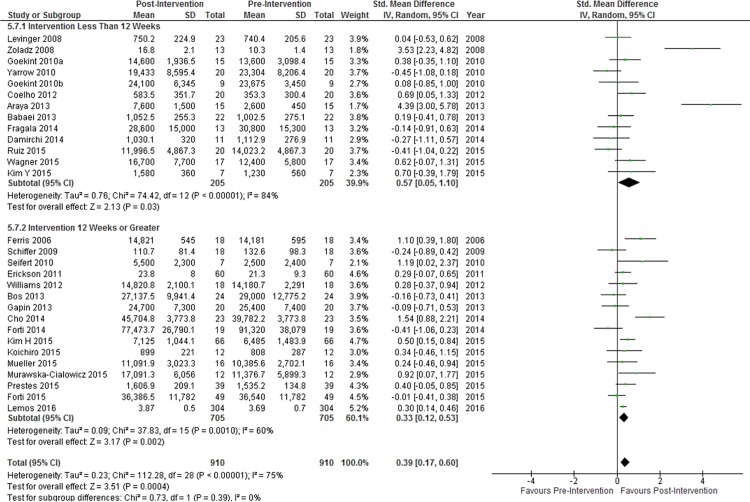
Subgroup analysis of studies with an intervention duration less than 12 weeks vs studies with an intervention duration of 12 weeks or greater. Diamonds indicate SMD and 95% CI. Test for subgroup differences: P = 0.39.

In meta-regression analyses, duration of exercise intervention (β = 0.038, p = 0.97, df = 28), exercise session time (β = 0.520, p = 0.61, df = 25), and number of exercise sessions per week (β = 1.471, p = 0.15, df = 27) were not significantly associated with changes in resting BDNF concentration (S2 Fig). Among studies in which exercise intensity was reported in percentage of VO_2Peak_ or percentage of maximum heart rate (n = 15), there was no association between exercise intensity and changes in resting BDNF concentration (β = 1.478, p = 0.16, df = 14). The median and modal exercise intervention duration was 12 weeks. Effect sizes in studies with an intervention duration of less than 12 weeks (n = 13) were not significantly different from effect sizes in studies with an intervention duration of 12 weeks or greater (Fig 4). Significant heterogeneity and inconsistency were observed in both subgroups (Fig 4).

#### Serum vs Plasma

**Fig 5:**
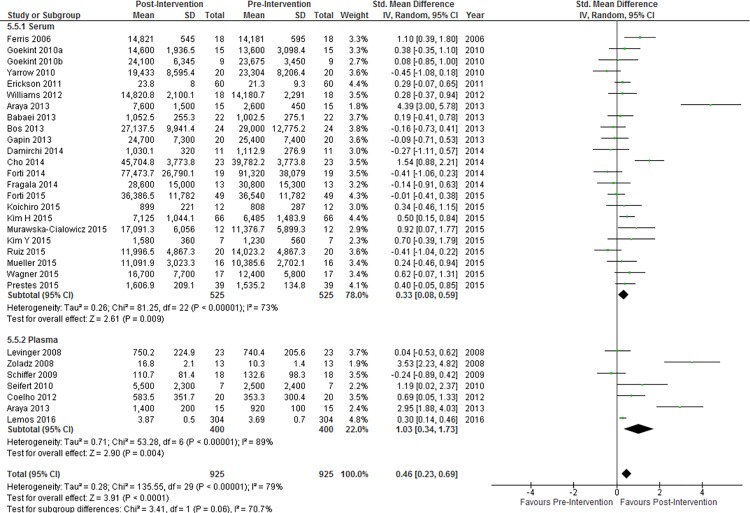
Subgroup analysis of BDNF concentration changes in serum vs plasma. Diamonds indicate SMD and 95% CI. Test for subgroup differences: P = 0.06.

BDNF was measured more commonly in serum than in plasma (Fig 5). Fig 5 shows subgroup differences in change in BDNF concentration after exercise between serum and plasma. The difference between these two subgroups was not significant.

#### Gender

**Fig 6:**
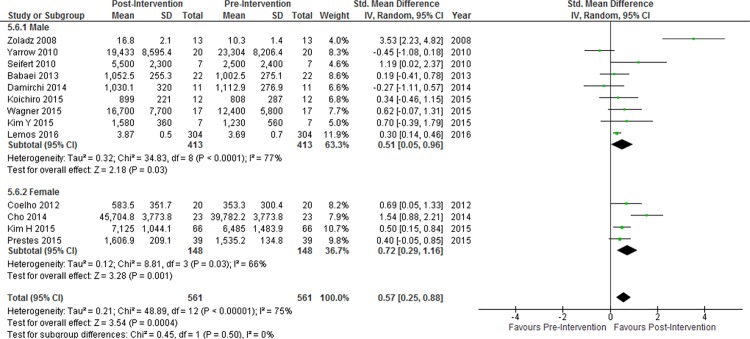
Subgroup analysis of difference in effect sizes between males and females. Diamonds indicate SMD and 95% CI. Test for subgroup differences: P = 0.50.

Nine studies included only men in their study population and four studies consisted entirely of females. Subgroup analysis showed no significant difference between effect sizes in studies that included only males and studies that included only females (Fig 6). Among studies in which the number of male and female participants was reported, no association was observed between percentage of male study participants and changes in resting BDNF concentration (β = -0.070, p = 0.95, df = 25).

#### Age and Body Mass Index (BMI)

The mean age of the study population was not associated with change in resting BDNF concentration (β = -1.182, p = 0.25, df = 27). Among studies reporting mean baseline BMI of participants, BMI was not associated with changes in resting BDNF concentration (β = 0.046, p = 0.96, df = 23).

## Discussion

Collectively, the evidence suggests an increase in resting peripheral BDNF concentrations after aerobic exercise training interventions but not after resistance training interventions. The increase in resting peripheral BDNF concentrations after an exercise intervention is heterogeneous and was found to have a small effect size [110]. This is important, as an increase in BDNF is proposed to be a mechanism through which physical activity enhances cognition and alleviates psychiatric symptoms [24, 25, 111–113]. This finding strengthens the evidence that aerobic exercise and resistance training constitute different physiological stimuli with respect to neurotrophin concentrations. For brain health, this finding suggests a basis for possible differential benefits from different exercise modalities. Furthermore, since BDNF is significantly lower in individuals with psychiatric disorders [26, 28–31], an increase in BDNF via exercise may confer clinical benefit by ameliorating this abnormality.

These results are similar to those reported in a previous meta-analysis on the effect of exercise on resting peripheral BDNF concentrations [72]. This current meta-analysis differs from that one due to the inclusion of 21 additional reports that were included due to a different search strategy and a later search period (February 2016). The larger number of included studies allowed for a more robust exploration of heterogeneity across studies and a stronger evaluation of current evidence.

As suggested by high Q^2^ and I^2^ values, heterogeneity across studies, as opposed to random sampling error, contributed considerably to variability in effect estimates. Inconsistency in effect estimates may be due to differences in study populations, exercise intervention characteristics, measurement techniques, and study quality. Our explorations of heterogeneity suggested that inconsistency was not significantly related to gender or age. This is important as exercise is recommended in elderly to promote brain health [114–116]. Similarly, exercise intervention duration, intensity and session time were not associated with degree of change in resting peripheral BDNF concentrations. As such, the current evidence could not determine an ideal exercise prescription for increasing resting peripheral BDNF concentrations.

No significant differences were found between studies measuring BDNF in serum versus plasma, although this subgroup analysis was limited by the relatively small number of studies measuring BDNF in plasma. As BDNF is stored in platelets, which are activated and release their contents in serum, serum concentrations of BDNF are substantially higher than plasma BDNF concentrations [67, 117]. Plasma BDNF concentration represents freely-floating, not stored, BDNF and thus may have a different physiological role from serum BDNF [118].

It is likely that there is a combination of different mechanisms through which physical activity confers benefits to neural health [12–14]. These may include increases in cerebral blood flow, changes in neuroendocrine responses, changes in endocannabinoid and neurotransmitter release, and structural changes in the central nervous system (CNS) [12, 113]. Increased BDNF is one proposed mechanism by physical activity confers these benefits [119]. Increased resting peripheral BDNF concentrations may indicate an increase in central BDNF production [120–123]. Increased BDNF production in the CNS may result in enhanced synaptogenesis and neuronal survival, resulting in structural changes and enhanced cognition [20, 22, 124–127]. While the findings support the hypothesis that BDNF concentration is increased in the blood after an exercise training intervention, it cannot be inferred that BDNF concentration is increased in the brain. Although one human study suggested an association between peripheral and central BDNF concentrations [121], the evidence in humans is limited. Since the human BBB is structurally and functionally different from those in animal models, we cannot infer from those models that BDNF can cross the human BBB [128, 129]. The roles of BDNF in the periphery are not well characterized and may involve regulation of energy homeostasis, modification of insulin activity, and modification of neuronal function in certain neuronal subpopulations in the peripheral nervous system [130–132].

Interestingly, a single nucleotide polymorphism (SNP) in the gene encoding BDNF, resulting in an amino acid substitution from valine to methionine, may impact the effect of exercise on BDNF concentrations [37, 133]. This SNP is present in approximately 30% of the global population and is associated with altered secretion of BDNF as well as a possible increase in serum BDNF concentration relative to those without the SNP [134–136]. The presence of this polymorphism may alter the relationship between fitness and brain outcomes such as cognition, mood, and response to mood treatments [133, 137, 138]. Few studies have assessed the potential impact of this SNP on the effect of exercise training on peripheral BDNF and brain outcomes. Findings from these reports suggest an increase in BDNF and improvements in memory after exercise only in those without the SNP [37, 133, 137]. However, those findings are limited by the small number of studies. Future work assessing the potential impact of this polymorphism on this effect and on the relationship between exercise and brain outcomes is required to determine whether exercise interacts equally with BDNF and brain outcomes in those with and without the polymorphism.

The large number of studies assessing the impact of exercise on resting concentrations of peripheral blood BDNF indicates the importance of this topic and allowed for a robust exploration of this effect. Nevertheless, this meta-analysis was limited by the heterogeneity in study populations and exercise interventions prescribed across different studies. A variety of populations and exercise interventions were included in this report, which we attempted to address via subgroup and meta-regression analyses. In addition, variable adherence to the intervention may have detracted from the quality of evidence.

Overall, this meta-analysis provides evidence for an increase in resting concentrations of peripheral blood BDNF after an exercise intervention. This effect is heterogeneous and may not be present in all individuals, suggesting the importance of further research to elucidate predictors of response. Interestingly, an increase in BDNF concentration may occur after aerobic exercise interventions but not resistance training interventions. Future studies in humans to determine whether peripheral concentrations of BDNF reflect central BDNF concentrations and whether BDNF can cross the human BBB are needed to assess the importance of these findings. In addition, further work to determine if changes in blood BDNF concentrations mediate clinical benefits, such as those on mood, cognition and other psychiatric disorders, would help to determine the usefulness of peripheral BDNF as a putative biomarker.

## Supporting Information

S1 Fig Funnel plot. (DOCX)

S2 Fig Meta-regression analyses graphs. (DOCX)

S1 Table Assessment of risk of bias and study reporting quality. + indicates yes;—indicates no;? indicates uncertain; NA indicates not applicable.
(XLSX)

S1 Text PRISMA checklist. (DOC)

S2 Text Sample search strategy (Medline database). (DOCX)
