# Supplementary figures and images for: The Effect of Exercise Training on Resting Concentrations of Peripheral Brain-Derived Neurotrophic Factor (BDNF): A Meta-Analysis
Source: PLoS One. 2016 Sep 22;11(9):e0163037. doi: 10.1371/journal.pone.0163037 (PMC5033477; doi:10.1371/journal.pone.0163037)

**Supplemental Figure 1. Funnel Plot**


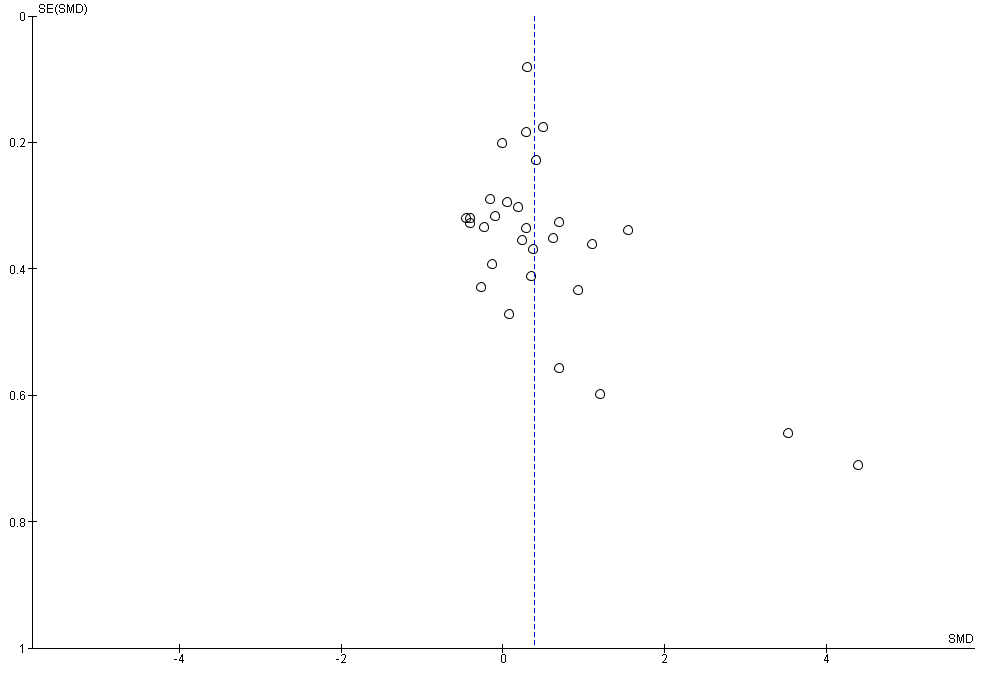

Supplement: S1 Fig — (DOCX) [file pone.0163037.s001.docx]
