# Supplementary material for: The Effect of Exercise Training on Resting Concentrations of Peripheral Brain-Derived Neurotrophic Factor (BDNF): A Meta-Analysis
Source: PLoS One. 2016 Sep 22;11(9):e0163037. doi: 10.1371/journal.pone.0163037 (PMC5033477; doi:10.1371/journal.pone.0163037)
Supplement: S2 Fig — (DOCX) [file pone.0163037.s002.docx]

**Supplemental Figure 2. Meta-regression analyses graphs**

*exercise session time in minutes

*exercise intensity measured as %VO_2 Peak_

*percentage of study participants that are male

*mean age of study participants

*mean BMI of study participants at baseline
