# Supplementary material for: The Effect of Exercise Training on Resting Concentrations of Peripheral Brain-Derived Neurotrophic Factor (BDNF): A Meta-Analysis
Source: PLoS One. 2016 Sep 22;11(9):e0163037. doi: 10.1371/journal.pone.0163037 (PMC5033477; doi:10.1371/journal.pone.0163037)
Supplement: S2 Text — (DOCX) [file pone.0163037.s005.docx]

**Supplemental Text 2. Sample search strategy (Medline database)**

1. Brain-Derived Neurotrophic Factor/
2. (brain?derived neurotrophic factor or BDNF).mp,hw,kw,nm.
3. 1 or 2
4. exp Exercise/
5. exp Exercise Therapy/
6. Exercise Test/
7. Physical Fitness/
8. exp Sports/
9. exp Exercise Movement Techniques/
10. Physical Exertion/
11. exp Physical Endurance/
12. exercise*.mp,hw,kw.
13. physical activit*.kw.
14. or/4-13
15. 3 and 14
